# Cuproptosis-related risk score based on machine learning algorithm predicts prognosis and characterizes tumor microenvironment in head and neck squamous carcinomas

**DOI:** 10.1038/s41598-023-38060-6

**Published:** 2023-07-22

**Authors:** Maodong Ye, Guangping Zhang, Yongjian Lu, Shuai Ren, Yingchang Ji

**Affiliations:** 1 Medical Cosmetic Center, First Affiliated Hospital of Shantou University Medical College, Shantou, 515041 Guangdong People’s Republic of China; 2 Shantou University Medical College, Shantou, 515041 Guangdong People’s Republic of China

**Keywords:** Immunology, Oncology, Risk factors

## Abstract

Cuproptosis is a recently discovered type of programmed cell death that shows significant potential in the diagnosis and treatment of cancer. It has important significance in the prognosis of HSNC. This study aims to construct a cuproptosis-related prognostic model and risk score through new data analysis methods such as machine learning algorithms for the prognosis analysis of HSNC. Protein–protein interaction network and machine learning methods were employed to identify hub genes that were used to construct a TreeGradientBoosting model for predicting overall survival. The relationship between the risk scores obtained from the model and features such as tumor microenvironment (TME) and tumor immunity was explored. The C-indexes of the TreeGradientBoosting model in the training and validation cohorts were 0.776 and 0.848, respectively. The nomogram based on risk scores and clinical features showed good performance, and distinguished the TME and immunity between high-risk and low-risk groups. The cuproptosis-associated risk score can be used to predict prognoses, TME, and tumor immunity of HNSC patients.

## Introduction

Head and neck squamous cell carcinoma (HNSC) is the seventh most common cancer worldwide^1^. The common symptoms of HNSC include persistent sore throat, difficulty or pain when swallowing, weight loss, and a lump in the throat or mouth^2^. The main risk factors for HNSC include ultraviolet radiation, tobacco use, alcohol consumption, chemical substances, and human papillomavirus infection. Long-term exposure to these risk factors can cause atypical hyperplasia or precancerous lesions in the skin and mucous membrane epithelium, eventually leading to HNSC. Current therapies for HNSC include surgery, chemotherapy, radiotherapy, and targeted immunotherapy^3^. Although most therapies improve long-term survival in patients, there are differences in overall survival (OS) among HNSC patients. The 5-year OS rate of advanced patients is only 50%^4^. Immunotherapy is a new treatment strategy for cancer, but many patients fail to respond to it. Currently, there are no biomarkers or scoring models that can accurately predict the prognosis of HNSC patients. Therefore, it is necessary to explore new prognostic models or risk scores for the analysis of prognosis and treatment outcomes in HNSC.

Cuproptosis is a type of programmed cell death induced by excessive accumulation of copper that was recently discovered by Tsvetkow et al.^5^. Copper is an essential element in various biological processes including mitochondrial respiration, and iron absorption. Excessive copper may lead to mitochondrial protein aggregation and cell death in different forms^6^. Several studies have shown that dysregulation of copper homeostasis plays a vital role in tumorigenesis and metastasis^7,8^, suggesting that cuproptosis has huge potential in cancer diagnosis and treatment.

Peng, Q et al.^9^ have found through multi-omics methods that cuproptosis-related genes may play an important role in the diagnosis, prognosis, immunotherapy and drug treatment prediction of HNSC. Tang S et al.^10^ have revealed the important significance of cuproptosis-related genes in the prognosis and tumor microenvironment of HSNC through bioinformatics methods. Although cuproptosis-related HNSC prognostic models have been constructed in his study, the models were mainly based on results of Cox proportional hazards analysis and only focused on the linear relation between cuproptosis-related genes and prognosis. Such models assume that only a linear correlation exists between the dependent and independent variables, which might oversimplify the complex relations (including non-linear associations and interactions^11,12^), resulting in poor predictive performance. Machine learning (ML) algorithms overcome this limitation by considering all possible interactions between the variables, providing an alternative for (semi-)parametric modeling. Gradient Boosting (GB), support vector machine (SVM), and random forest (RF) algorithms are machine learning algorithms that are well-known for their interpretability, lack of overfitting, and ease of use^13^. Machine learning algorithm has achieved many successes in its application to the fields of medicine and biology. For example, it has been used to predict the survival outcomes of triple-negative breast cancer^14^, the recurrence of colorectal cancer^15^, and the prognostic analysis of glioma^16^. These studies have utilized common clinical variables to construct non-linear models, which have demonstrated stronger predictive performance than linear models. The aim of this study was to use machine learning algorithms to construct a better cuproptosis-related model and risk score that could predict the prognosis and tumor characteristics of patients for use in clinical practice.

## Materials and methods

### Data acquisition

TCGA-HNSC mRNA sequencing data and related clinical information were downloaded from The Cancer Genome Atlas (TCGA) database. GSE41613 gene expression data and sample information were retrieved from Gene Expression Omnibus (GEO). RNA-seq data from TCGA were normalized as transcripts per thousand base million (TPM).

### Construction and validation of cuproptosis-related risk score based on machine learning method

A previous study^5^, identified 347 potential cuproptosis-associated genes through full-genome loss of function CRISPR-Cas9 screens. The “survival” package in R was used to carry out univariate Cox analysis to determine the relationship between expression of the 347 genes and survival based on the TCGA-HNSC dataset. From this analysis, 55 cuproptosis-associated genes with prognostic potential for HNSC were identified (*P* < 0.05). Protein–protein interaction (PPI) network analysis was performed using the STRING online website (https://string-db.org/), and the results were imported to Cytoscape V3.9.1. The hub genes network was then extracted using the CytoHubba Cytoscape plugin. The 20 genes with the highest score (maximum correlation criterion, MCC) were selected as hub genes for further investigation.

The 20 hub genes were used to establish four machine-learning models (SVM model, RF model, TreeGradientBoosting model, and ComponentwiseGradientBoosting model) and a conventional model, Cox proportional hazards model, for predicting OS. Harrell's concordance index (C-index) and the area under the receiver operating characteristic curve (AUC) were used to evaluate the efficiency of the models in predicting prognosis. The model with the highest C-index was identified as the optimal model.

The importance of each hub gene in the model was assessed based on the optimal model. We evaluate the significance of each hub gene by determining its impact on the C-index. This is done by measuring the decrease in the C-index after disrupting the association between this feature and survival. The process of evaluating the feature importance of the hub gene was achieved using the ‘PermutationImportance’ function in the Python module ‘eli5.sklearn’. The parameters for this function are set to n_iter = 5 and random_state = 20. Features with weight greater than 0.015 are considered crucial to the model’s predictive performance. The genes that contributed most to the prognostic model were used to establish another TreeGradientBoosting model. TCGA-HNSC and the GSE41613 datasets served as the training and validation cohorts, respectively. The prognostic model was uploaded to GitHub (https://github.com/dongbianrichu/HCC-prognosis-predictive-model.git). The predicted score of the 10-gene-TreeGradientBoosting model was multiplied by 10 to obtain the risk score. Cases were categorized into high-risk and low-risk groups using the median risk score as the cutoff.

### Machine learning models construction

SVM is a supervised machine learning algorithm that can be used for survival analysis. It works by finding a hyperplane that separates the data points into different classes. Meanwhile, Random Forest is an ensemble learning method that operates by constructing multiple decision trees during training time. Gradient Boosting is a machine learning technique that can be applied to survival analysis. In this case, we used component-wise least squares and survival decision trees as two types of base learners. The Gradient Boosting algorithm generates a different weak prediction model at each step and combines them into a final model with different weights. The predictions of the weak models produced by Gradient Boosting at each step generate a unanimous gradient direction for the loss function.

We constructed machine learning models using the scikit-survival module (version 0.12.1), including RF, Tree GB, and Component GB, in Python (version 3.7). Cox model was also constructed for comparison with the ML models as a conventional model. These machine learning algorithms have many hyperparameters that can significantly impact prediction performance. We used grid search to determine the optimal combination of hyperparameters. The modules in Python and hyperparameters for grid search were listed in Supplementary Table 1. During each cross-validation, we randomly excluded 1/3 of the data in the training set (TCGA-HNSC dataset) as out-of-bag (OOB) data for validation. We calculated the mean C-index on the validation data for different combinations of hyperparameters after performing cross-validation 50 times. The combination of hyperparameters with the best C-index was selected as the optimum. The process of grid search was achieved using the ‘GridSearchCV’ function in the Python module ‘sklearn.model_selection’. The optimal parameters for constructing each model were presented in Supplementary Table 1. Besides, the parameters of the second TreeGradientBoosting model was consistent with the first.

### Survival analysis and pathway enrichment

The Kaplan−Meier curves were plotted using the R package “survminer”. Multivariate Cox analysis was performed based on risk score, gender, pathologic stage, and age, and the results were used to plot a nomogram with the “rms” package in R to predict 1-, 3-, and 5-year OS. ROC curves were plotted to evaluate its performance. Gene Ontology (GO) and Kyoto Encyclopedia of Genes and Genomes (KEGG) enrichment analyses were carried out for differentially-expressed genes between the high-risk and low-risk groups using the “clusterProfiler” package^17^. The stemness indexes (SI) were calculated according to the model by Malta TM et al.^18^.

### Gene mutation and copy number variation (CNV)

Gene mutation data were retrieved from Genomic Data Commons (GDC; https://portal.gdc.cancer.gov/) and analyzed using the “maftools” package. CNV data were retrieved from the UCSC Xena database (http://xena.ucsc.edu/). The CNV in high-risk and low-risk groups were analyzed using the "RCircos" package in R.

### Tumor immunity-associated analysis and antitumor drug analysis

The data of six immune cell subtypes were retrieved from the study conducted by Tamborero et al.^19^. Immune cell infiltration data were acquired from the TIMER 2.0 database (http://timer.cistrome.org/) and evaluated using CIBERSORT, MCPCOUNTER, QUANTISEQ, and XCELL algorithms. Immune-related pathway enrichment was evaluated using the “GSVA” package in R. Bagaev et al.^20^ proposed 29 functional gene expression signatures (Fegs) to represent cells and functions of the tumor microenvironment (TME). The microsatellite instability (MSI) of each sample was calculated using the “PreMSIm” package in R^21^, and the samples classified into MSI-high (MSI-H) and MSI-low/microsatellite stability (MSS) based on the results. The efficacy of immune checkpoint inhibitors (ICI) in the high-risk and low-risk groups was assessed using Tumor Immune Dysfunction and Exclusion^22^ (TIDE, http://tide.dfci.harvard.edu/login/). Xu et al.^23^ defined antitumor immune response as a series of progressive processes. In this study, we also explored the correlation between risk score and each process. The antitumor drug sensitivity of each sample was predicted based on gene expression data using the R package "oncoPredict".

### Statistical analysis

TCGA-HNSC and GSE41613 data were all preprocessed and normalized before model construction. Wilcoxon test, log-rank analysis, Delong’s test and the "compareC" package in R were used to compare continuous variables, survival rates, AUCs and C-indexes, respectively, between groups. Correlation analysis was carried out using Pearson or Spearman analysis. *P* < 0.05 was regarded as statistically significant (* *p* < 0.05; ** *p* < 0.01; *** *p* < 0.001; **** *p* < 0.0001).

## Results

### Gene screening, model construction, and risk score generation

**Figure 1:**
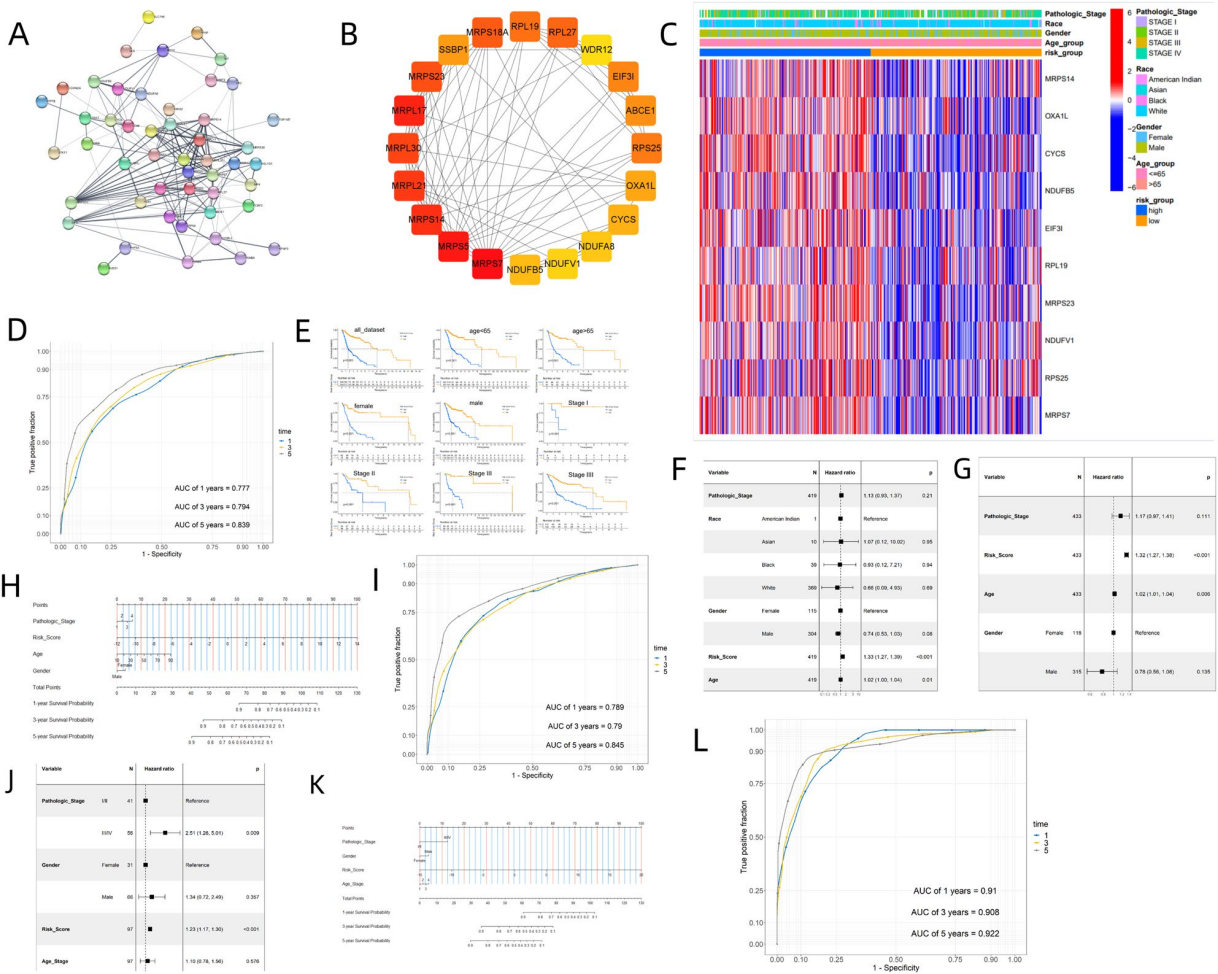
(**A**) PPI network of 55 cuproptosis-related genes significant in Cox regression analysis. (**B**) PPI network of top 20 hub genes in MCC. (**C**) ROC curves of 1-, 3-, and 5-year survival of 10-gene-TreeGradientBoosting model using the TCGA-NHSC database. (**D**) Heatmap showing the distribution of high-risk and low-risk groups and clinical features in the TCGA-NHSC database. (**E**) Kaplan − Meier plot and results of stratified analysis of high-risk and low-risk groups in the TCGA-NHSC database. (**F**) Forest plot showing multivariate Cox regression analysis of risk score and all clinical features in the TCGA-NHSC dataset. (**G**) Forest plot depicting multivariate Cox regression analysis of risk score and filtered clinical features in the TCGA-NHSC dataset. (**H**) Nomogram using risk score and clinical features in the TCGA-NHSC dataset. (**I**) ROC curves of the nomogram predicting 1-, 3-, and 5-year survival. (**J**) Forest plot showing multivariate Cox regression analysis of risk score and all clinical features in the TCGA-NHSC dataset. (**K**) Nomogram using risk score and clinical features in the TCGA-NHSC dataset. (**L**) ROC curves of the nomogram predicting 1-, 3-, and 5-year survival. The images was generated by R for Windows (version: V4.0.3, URL: https://www.r-project.org/).

Univariate Cox regression analysis conducted to determine the relationship between expression of 347 cuproptosis-related genes and survival, identified 55 genes that were significantly associated with survival. The HR, 95% CI, and *P*-values of these genes are presented in Supplementary Table 2. Figure 1A shows the PPI network of the 55 genes. The top 20 hub genes in MCC were selected, and their MCC scores and ranks are shown in Supplementary Table 3, and their PPI network are shown in Fig. 1B. The 20 hub genes were used to establish 4 machine-learning prognostic models and a conventional Cox prognostic model. The C-indexes of SVM, RandomForest, TreeGradientBoosting, ComponentwiseGradientBoosting and Cox models were 0.595, 0.747, 0.819, 0.568 and 0.665 respectively (Table 1). Comparison of the C-indexes among the five models showed that the TreeGradientBoosting model performed better than other models (Table 2, *P* < 5.0 × 10^–2^). RandomForest and TreeGradientBoosting models performed well in predicting 1-, 3-, and 5-year survival with AUCs > 0.7 (Supplementary Table 4). The comparison results of the AUC of five models for different survival periods are shown in Supplementary Table 5, and they indicate that the TreeGradientBoosting model had the best performance (*P* < 5.0 × 10^–2^). From the perspective of the C-index and the AUC of the prediction of different survival periods, the RandomForest and TreeGradientBoosting models in machine learning models perform better than the conventional Cox model. We assessed the importance of each gene in predicting survival using the TreeGradientBoosting model (Supplementary Table 6). The genes with weight greater than 0.015 were MRPS14, OXA1L, CYCS, NDUFB5, EIF3I, RPL19, MRPS23, NDUFV1, RPS25, and MRPS7. These top 10 genes were used to construct the second TreeGradientBoosting model. The C-index of the 10-gene-TreeGradientBoosting prognostic model in the TCGA dataset was 0.776, and the AUCs for predicting 1-, 3-, and 5-year survival were 0.777, 0.794, and 0.839, respectively (Fig. 1D).

**Table 1:** The C-index and 95% CI of different OS predictive models.

	C-index	95% CI
Fast survival SVM	0.595	0.557–0.633
Random survival forest	0.747	0.714–0.780
Tree gradient boosting	0.819	0.793–0.847
Componentwise gradient boosting	0.568	0.527–0.608
Cox PH survival	0.665	0.629–0.690

**Table 2:** The comparison on C-index between different OS predictive models.

	*P*-value
Fast survival SVM versus random survival forest	< 0.001
Fast survival SVM versus tree gradient boosting	< 0.001
Fast survival SVM versus componentwise gradient boosting	0.040
Fast survival SVM versus cox PH survival	0.112
Random survival forest versus tree gradient boosting	< 0.001
Random survival forest versus Componentwise gradient boosting	< 0.001
Random survival forest versus cox PH survival	0.002
Tree gradient boosting versus Componentwise gradient boosting	< 0.001
Tree gradient boosting versus cox PH survival	< 0.001
Componentwise gradient boosting versus cox PH survival	< 0.001

Patients were assigned to high-risk and low-risk groups using the median risk score as the cutoff. There was a significant difference in expression of cuproptosis-related genes between high-risk and low-risk groups (Fig. 1C). K-M analysis in the TCGA database showed a significant difference in survival between high-risk and low-risk groups (Fig. 1E). Further stratified analysis revealed the extensive discriminatory ability of the model (Fig. 1E). The multivariate Cox analysis illustrated that risk group was a prognostic factor independent of clinical features (e.g., pathologic stage, age, gender, and race) (Fig. 1F). A nomogram (Fig. 1H) consisting of risk score, gender, pathologic stage, and age was plotted based on the results of multivariate Cox analysis (Fig. 1G). The C-index of the nomogram was 0.776, and the AUCs of 1-, 3-, and 5-year survival were 0.789, 0.790, and 0.845, respectively (Fig. 1I), which indicated good performance. The GEO dataset was used as the validation cohort, in which the C-index of the model was 0.848. Multivariate Cox analysis verified that risk score was an independent factor for prognosis (Fig. 1J). The nomogram (Fig. 1K) based on the results of multivariate Cox analysis had a C-index of 0.854, and the AUCs of 1-, 3-, and 5-year survival were 0.910, 0.908, and 0.922, respectively (Fig. 1L).

### Genetic alterations

**Figure 2:**
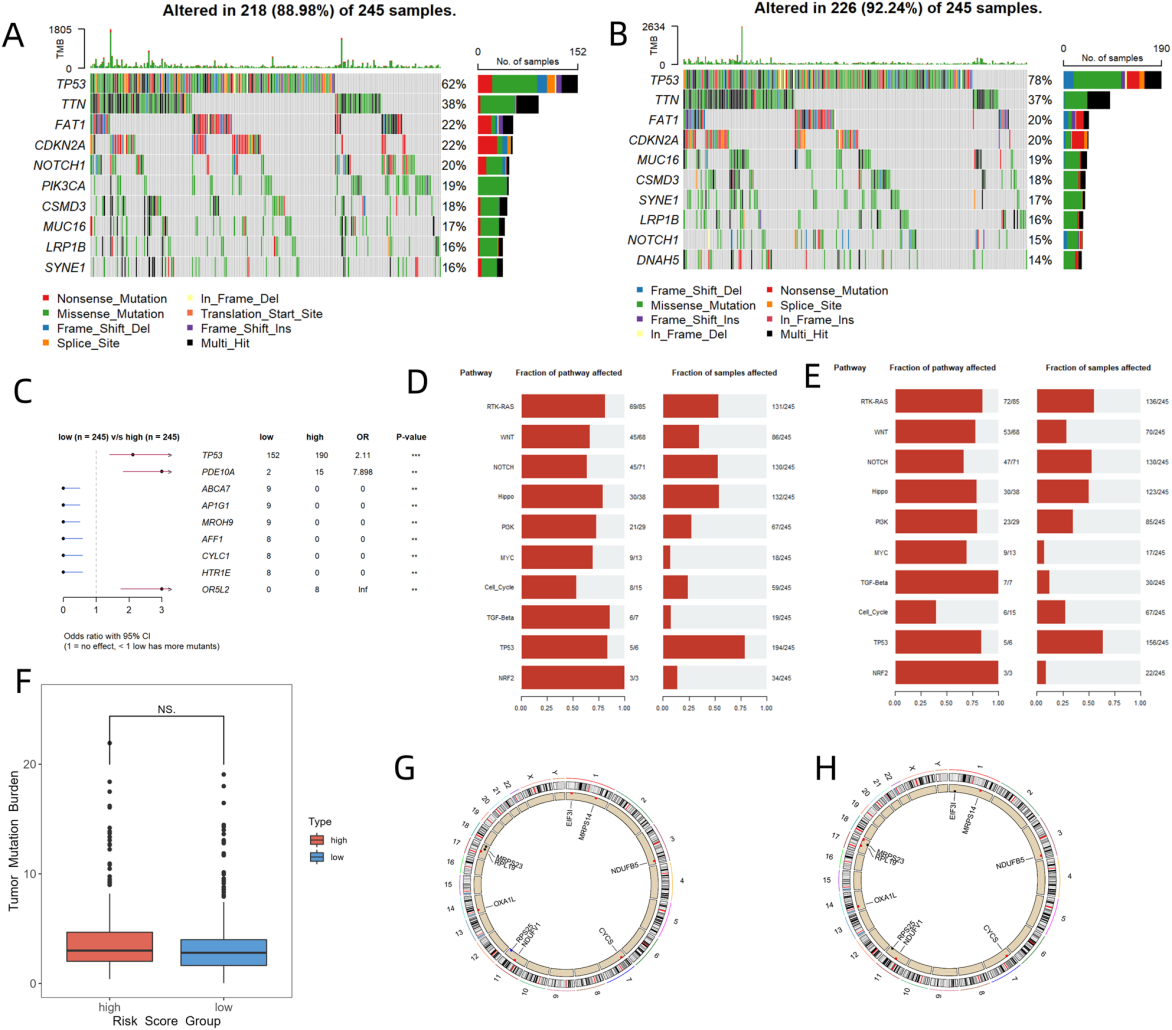
(**A**) Oncoplots showing mutated genes in the low-risk group from the TCGA-NHSC dataset. (**B**) Oncoplots showing mutated genes in the high-risk group. (**C**) Forest plot showing the difference in somatic mutations between the two groups. (**D**) Enrichment of tumor-related signaling pathways of mutated genes in the high-risk group. (**E**) Enrichment of tumor-related signaling pathways of mutated genes in the low-risk group. (**F**) Box plot exhibiting the difference in tumor mutation load between the high-risk and low-risk groups. (**G**) Circos plot showing CNV in the high-risk group. (H) Circos plot showing CNV in the low-risk group. The images was generated by R for Windows (version: V4.0.3, URL: https://www.r-project.org/). (The *P* values were shown as **p < 0.01, ***p < 0.001. ns for not significant).

We also compared gene mutations such as somatic mutations and CNV between the two risk groups of TCGA-HNSC. The waterfall plots of mutated genes are presented in Fig. 2A,B. The occurrence of somatic mutations was 88.98% in the low-risk group, which was lower than the 92.24% recorded in the high-risk group. The 4 most frequently mutated genes in the two groups included TP53, TTN, FAT1, and CDKN2A. Figure 2C shows the difference in somatic mutations between the two groups. The high-risk group had significantly higher TP53 (*P* < 0.001) and PDE10A (*P* < 0.01) mutation frequency than the low-risk group, while the low-risk group had higher ABCA7 (*P* < 0.01), AP1G1 (*P* < 0.01), MROH9 (*P* < 0.01), and AFF1 (*P* < 0.01) mutation frequency than the high-risk group. The top 4 tumor-associated signaling pathways that were enriched in high-risk and low-risk groups were similar and consisted of TP53, RTK-RAS, NOTCH, and Hippo signaling pathways (Fig. 2D,E). There was no significant difference in tumor mutation load between the two groups (Fig. 2F). The circos plots of CNV are shown in Fig. 2G,H.

### Functional enrichment analysis

**Figure 3:**
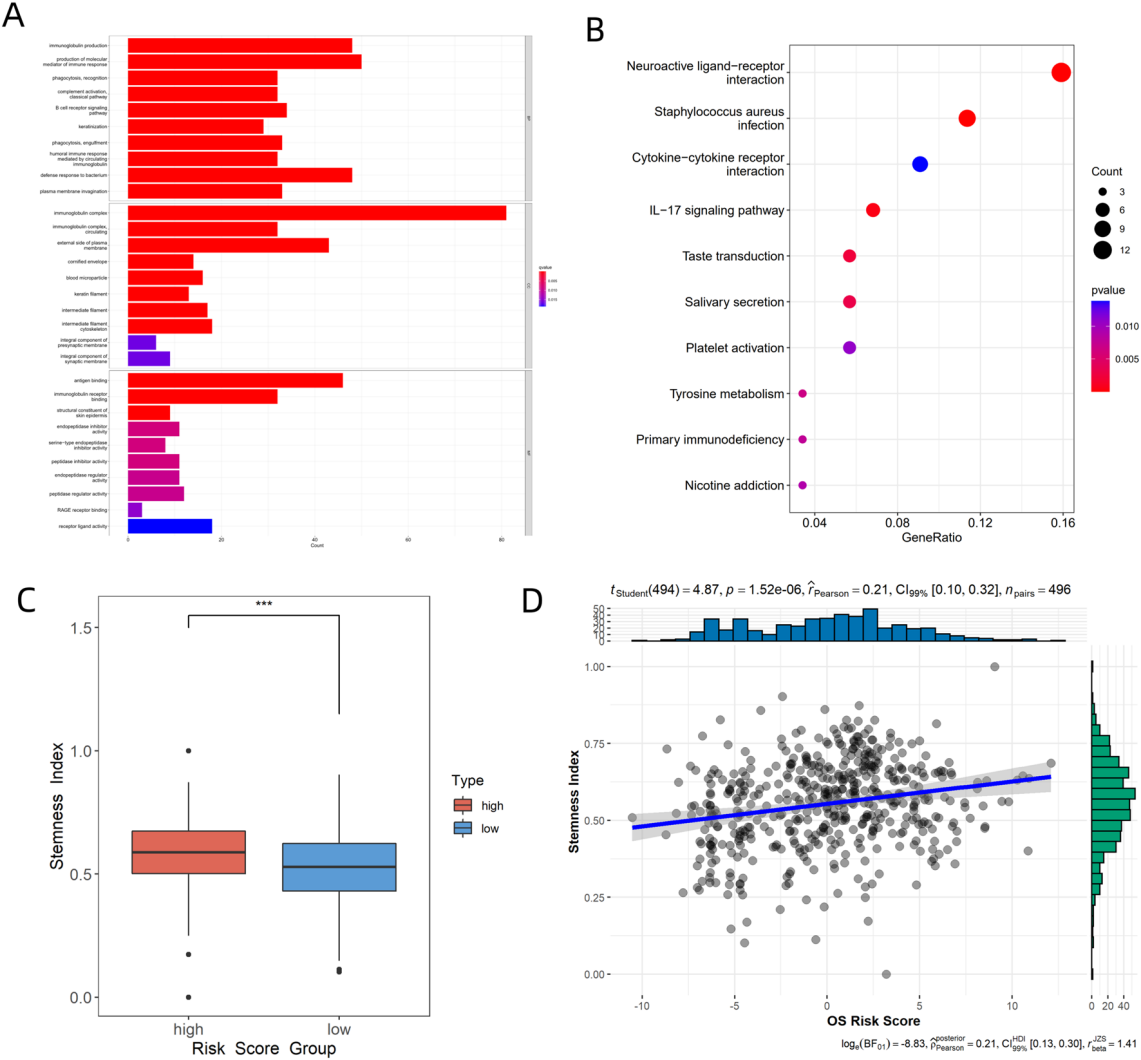
(**A**) Barplot showing results of GO enrichment analysis of the high-risk and low-risk groups. (**B**) Bubble plot displaying results of KEGG enrichment analysis of high-risk and low-risk groups^1–3^. (**C**) Differences in stemness index between high-risk and low-risk groups. (**D**) Scatter diagram showing correlation between risk score and stemness index. The images was generated by R for Windows (version: V4.0.3, URL: https://www.r-project.org/). (***p < 0.001) ^1^ Kanehisa, M. and Goto, S.; KEGG: Kyoto Encyclopedia of Genes and Genomes. Nucleic Acids Res. 28, 27–30 (2000). ^2^ Kanehisa, M; Toward understanding the origin and evolution of cellular organisms. Protein Sci. 28, 1947–1951 (2019). ^3^ Kanehisa, M., Furumichi, M., Sato, Y., Kawashima, M. and Ishiguro-Watanabe, M.; KEGG for taxonomy-based analysis of pathways and genomes. Nucleic Acids Res. 51, D587-D592 (2023).

The relevant pathways were analyzed using the GO and KEGG databases to further investigate the underlying mechanism. From GO analysis, the main biological processes identified were immunoglobulin production, production of molecular mediators of the immune response, phagocytosis, complement activation, and B cell receptor signaling pathway (Fig. 3A). KEGG pathway enrichment analysis identified neuroactive ligand-receptor interaction, cytokine-cytokine receptor interaction, and IL-17 signaling pathway as the main pathways (Fig. 3B). We also found that the stemness index was positively correlated with the risk score (r = 0.21, *P* = 1.5 × 10^–6^, Fig. 3C), and that the SI of the high-risk group was significantly higher than that of the low-risk group (*P* < 1.0 × 10^–3^, Fig. 3D).

### Immune landscapes of different groups

**Figure 4:**
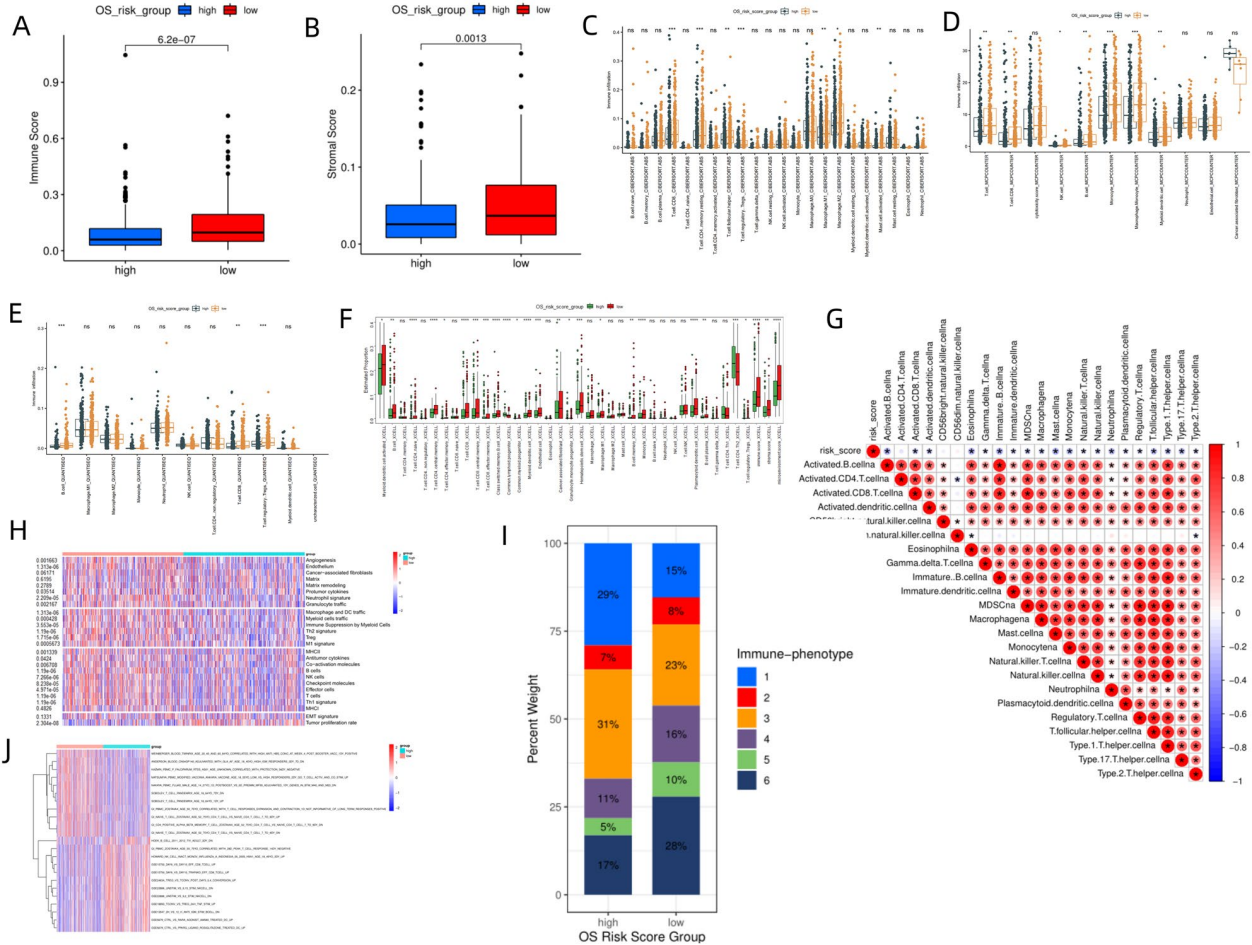
(**A**) Differences in immune scores between high-risk and low-risk groups from TCGA-NHSC dataset. (**B**) Differences in stromal scores between the two groups. (**C**) Boxplot showing the difference in immune cell infiltration between high-risk and low-risk groups using the CIBERSORT.ABS algorithm. (**D**) Differences in immune cell infiltration between two groups using the MCPCOUNTER algorithm. (**E**) Differences in immune cell infiltration between two groups using the QUANTISEQ algorithm. (**F**) Differences in immune cell infiltration between two groups using the XCELL algorithm. (**G**) Correlation coefficients of risk scores and various immune cells. (**H**) Heatmap showing the difference in 29 Fegs between high-risk and low-risk groups. (**I**) Comparison of 6 immune subtypes between two groups. (**J**) Heatmap showing differences in immune-related pathway enrichment between high-risk and low-risk groups. The images was generated by R for Windows (version: V4.0.3, URL: https://www.r-project.org/). (The *P* values were shown as **p < 0.01, ***p < 0.001. ns for not significant).

TME is a complex environment consisting of tumor cells, immune cells, and stromal cells that affects tumor cell infiltration and metabolism^24^. ESTIMATE analysis revealed that the high-risk group had a significantly lower immune score than the low-risk group (*P* < 0.0001, Fig. 4A), indicating that the low-risk group had a higher degree of immune cell infiltration. The high-risk group had a significantly lower stromal score than the low-risk group (*P* = 0.0013, Fig. 4B). Analysis of immune cell infiltration level using CIBERSORT.ABS algorithm revealed that the high-risk group had a high abundance of M1 macrophages (*P* < 0.01), M2 macrophages (*P* < 0.05), mast cells (*P* < 0.05), resting CD4 + memory T cells (*P* < 0.001), CD8 + T cells (*P* < 0.001), T follicular helper cells (*P* < 0.01), and regulatory T cells (*P* < 0.001) than the low-risk group (Fig. 4C). The value of M1 macrophages/M2 macrophages in the high-risk group was 0.4673427, while M1/M2 in the low-risk group was 0.4901091 (Fig. 4C), indicating that the high-risk group was more prone to tumor immunosuppression. Figure 4D shows the results of immune cell infiltration assessment using the MCPCOUNTER algorithm. The low-risk group had higher infiltration of T cells (*P* < 0.01), CD8 + T cells (*P* < 0.01), NK cells (*P* < 0.05), B cells (*P* < 0.01), monocytes (*P* < 0.001), macrophages (*P* < 0.001), and myeloid dendritic cells (*P* < 0.01). Results of QUANTISEQ algorithm showed that the low-risk group had high infiltration of B cells (*P* < 0.001), CD8 + T cells (*P* < 0.01), and regulatory T cells (*P* < 0.001) (Fig. 4E). Figure 4F shows the results of the XCELL algorithm. Correlation analysis between immune cell infiltration and risk score using ssGSEA algorithm revealed that the risk score was negatively associated with the score of 20 kinds of immune cells, including CD4 + T cells and CD8 + T cells (Fig. 4G). Comparison of the scores of 29 Fegs between the high-risk and low-risk groups, revealed that the low-risk group had significantly higher antitumor immune marker levels than the high-risk group, including antitumor cytokines, co-activation molecule, checkpoint molecules, and T cells (Fig. 4H). Tamborero et al.^19^ proposed classification of tumor cells into immunophenotypes 1–6, with immunophenotypes 1–2 representing low cytotoxicity, immunophenotypes 3–4 representing medium cytotoxicity, and immunophenotypes 5–6 representing high cytotoxicity. Immunophenotypes with low and medium cytotoxicity are associated with disruption of tumor immunity, which promotes immune evasion. The high-risk group (36%, 42%) had a higher percentage of immunophenotypes with low and medium cytotoxicity than the low-risk group (23%, 39%), and a lower percentage of high cytotoxicity immunophenotypes than the low-risk group (22 vs. 38%, Fig. 4I). The immune-related pathway enrichment was also compared between the high-risk and low-risk groups (Fig. 4J).

### Response to immunotherapies

**Figure 5:**
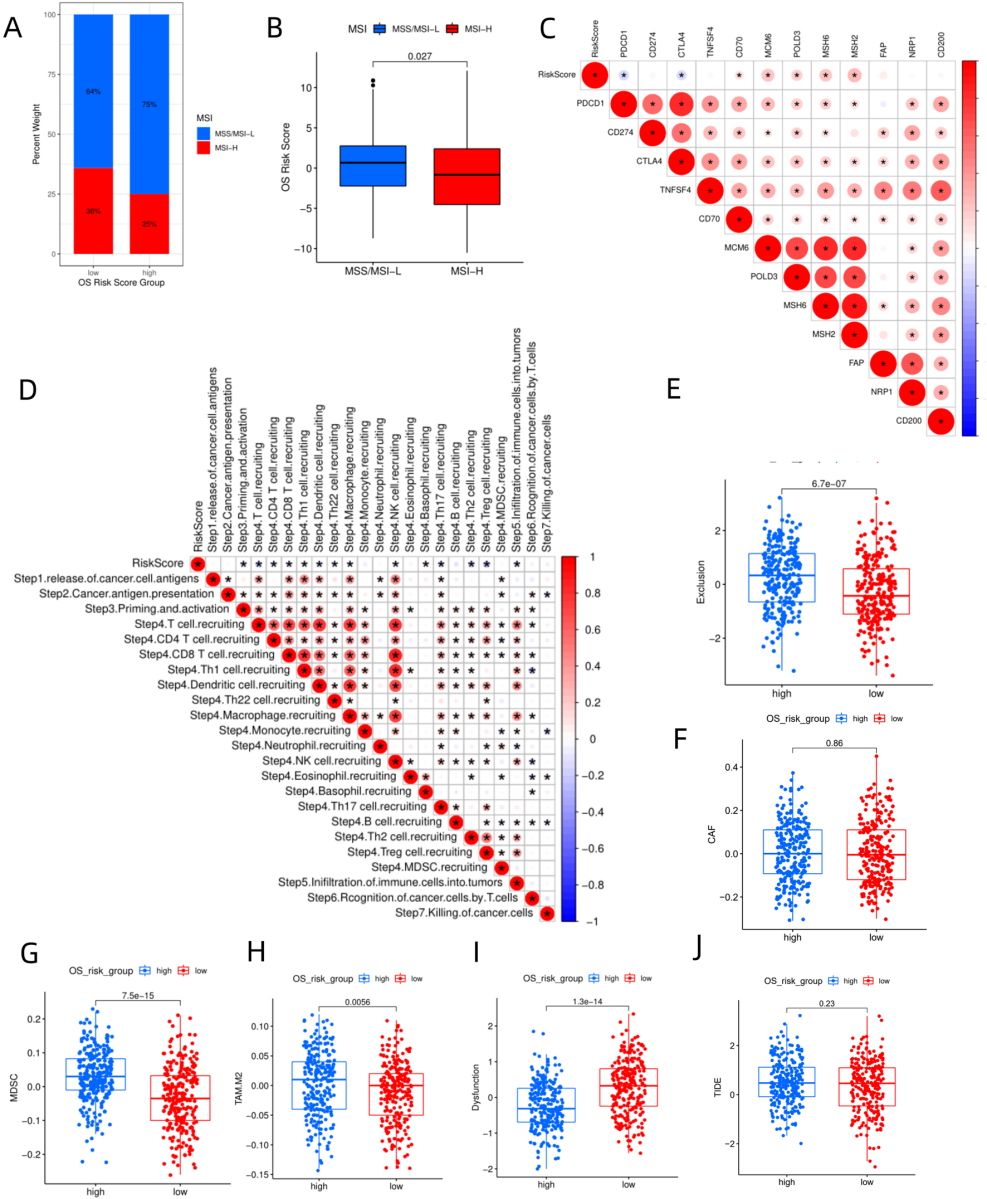
(**A**) Distribution of MSI in high-risk and low-risk groups from the TCGA-NHSC dataset. (**B**) Comparison of risk scores of MSI-high and MSI-low/microsatellite stability. (**C**) Correlation coefficient diagram between risk scores and 12 immune checkpoint genes. (**D**) Correlation coefficient diagram between risk scores and immune activity scores of each step of the Cancer-Immunity Cycle. (**E**) Boxplot illustrating the difference in Exclusion scores between high-risk and low-risk groups. (**F**) Difference in cancer-associated fibroblasts (CAF) scores between high-risk and low-risk groups. (G) Differences in CAF scores between high-risk and low-risk groups. (**H**) Difference in M2 tumor-associated macrophages (TAM) scores between two groups. (**I**) Differences in Dysfunction scores between the two groups. (**J**) Differences in TIDE scores between the two groups. The images was generated by R for Windows (version: V4.0.3, URL: https://www.r-project.org/).

As for microsatellite instability, the low-risk group had lower MSS/MSI-L (64 vs. 75%) and higher MSI-H (36 vs. 25%) than the high-risk group (Fig. 5A), and the risk score of MSI-H was significantly higher than that of MSS/MSI-L (*P* = 0.027, Fig. 5B). Correlation analysis between risk score and 12 immune checkpoint genes (Fig. 5C) showed that immune checkpoint genes PDCD1 and CTLA4 were negatively associated with risk scores (*P* < 0.05), while CD274 had no significant association with risk scores. These results demonstrated that lower risk scores were associated with higher efficacy of immunosuppressive agents. Risk scores were positively correlated with genes such as MCM6, POLD3, MSH6, and MSH2 (Fig. 5C), but negatively correlated with 14 steps in Cancer-Immunity Cycle (Fig. 5D), including initiation activation, T cell recruitment, CD4+ T cell recruitment, CD8+ T cell recruitment, Th1 cell recruitment, and NK cell recruitment. We also observed that the high-risk group had higher scores of myeloid-derived suppressor cells (MDSCs, Fig. 5G) and tumor-associated M2 macrophages (TAM) (Fig. 5H), as well as a higher Exclusion score (Fig. 5E). The difference on cancer associated fibroblasts (CAFs) score (Fig. 5F) between the two groups wasn’t significant. Although the high-risk group had a lower Dysfunction score than the low-risk group (Fig. 5I), its TIDE score was higher than that of the low-risk group (Fig. 5j *P* > 0.05).

### Antitumor drug sensitivity

**Figure 6:**
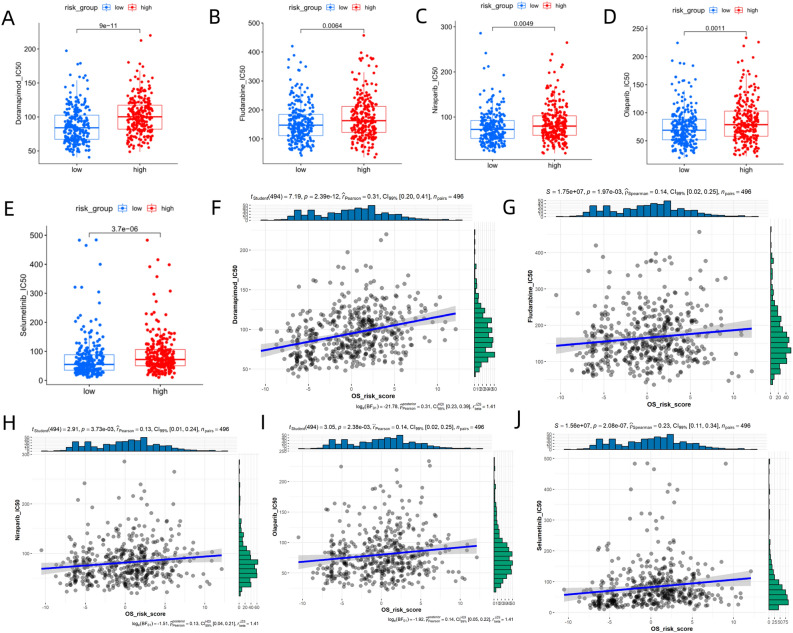
(**A**) Boxplot demonstrating differences in Doramapimod IC50 between high-risk and low-risk groups from the TCGA-NHSC dataset. (**B**) Differences in Fludarabine IC50 between high-risk and low-risk groups. (**C**) Differences in Niraparib IC50 between high-risk and low-risk groups. (**D**) Differences in Olaparib IC50 between high-risk and low-risk groups. (**E**) Differences in Selumetinib IC50 between high-risk and low-risk groups. (**F**) Scatter diagram showing the correlation between risk score and Doramapimod IC50. (**G**) Correlation between risk scores and Fludarabine IC50. (**H**) Correlation between risk scores and Niraparib IC50. (**I**) Correlation between risk scores and Olaparib IC50. (**J**) Correlation between risk scores and Selumetinib IC50. The images was generated by R for Windows (version: V4.0.3, URL: https://www.r-project.org/).

Chemotherapy and targeted therapy are common treatments for HNSC. The "oncoPredict" package in R was used to predict the sensitivity of TCGA-HNSC patients to the chemotherapeutic agent (Fludarabine), p38 MAPK inhibitor (Doramapimod), targeted therapy agent (Selumetinib and Olaparib), and PARP inhibitor (Niraparib). The half-maximal inhibitory concentrations (IC50) of the low-risk group were significantly lower than those of the high-risk group (Fig. 6A–E), with Doramapimod showing the most significant difference between the two groups (Fig. 6A). The IC50 of these 5 drugs were positively correlated to the risk score (Fig. 6F–J). Although these 5 drugs are not standard drugs for HNSC, they all have good therapeutic potential.

## Discussion

Copper is a trace element that plays significant roles in physiological activities, such as free radical scavenging, mitochondrial respiration, redox chemistry, and cross-linked elastin^25^. Copper homeostasis is important in the proper functioning of various physical and biological processes. Copper homeostasis imbalance is closely correlated with various diseases^26^. Evidence from previous studies has demonstrated that copper is critical in tumor cell proliferation, angiogenesis, and metastasis^27^. A recent study by Peter Tsvetkov et al. reported a new form of programmed cell death, called cuproptosis, which is induced by excessive copper accumulation. Several bioinformatics studies have reported that there is significant correlation between cuproptosis and TME and tumor immunity, indicating the significance of cuproptosis in predicting prognosis and response to immunotherapy^28^. Therefore, cuproptosis has potential application in HNSC. In this study, bioinformatics and machine learning techniques were used to construct a cuproptosis-associated 10-gene-TreeGradientBoosting model for predicting HNSC prognosis with corresponding risk scores generated. We found that the risk scores were correlated with tumor features such as TME and tumor immunity.

Four machine-learning models and a conventional Cox model were established in this study. From the perspective of the C-index and the AUC of the prediction of different survival periods, the RandomForest and TreeGradientBoosting models in machine learning models perform better than the conventional Cox model. This is primarily due to the fact that the Cox model is a type of generalized linear model that handles variables based on the proportional hazards assumption, and directly disregards the intricate relationship between the dependent and independent variables during the modeling process. In contrast, RandomForest and TreeGradientBoosting do not assume a linear relationship and have explored the nonlinear associations among various variables to some extent, thus bringing them closer to the complex reality of the world. What’s more, the Gradient Boosting algorithm is a type of ensemble machine-learning method that involves the addition of multiple weak classifiers with different weightings, and integrates the linear and non-linear relationships between independent and dependent variables. Since Gradient Boosting is an ML algorithm framework, its predictive performance varies with different base learners. In this study, we used survival decision tree and component-wise least squares as base learners. Currently, GradientBoosting is extensively applied in medical predictive modeling and has been remarkable in predicting the prognoses of neuroblastoma^29^, leukemia, and breast cancer. In this study, the TreeGradientBoosting model achieved the best predictive performance. Both TreeGradientBoosting and RandomForest are ensemble models based on survival decision trees. However, unlike RandomForest where each decision tree has the same weight, TreeGradientBoosting adjusts the weight of each decision tree based on feedback errors during the model construction process. This boosting modeling strategy of TreeGradientBoosting determines its superiority over RandomForest which uses bagging as its modeling strategy. The difference between TreeGradientBoosting and ComponentwiseGradientBoosting is that ComponentwiseGradientBoosting uses component-wise least squares as base learners. Component-wise least squares is a statistical method that is a variant of the least squares method and can be considered as a linear regression model to some extent. In contrast, TreeGradientBoosting abandons the linear assumption to a greater extent, so it has better prediction performance for complex relationships. The optimal model, the TreeGradientBoosting model, was used to calculate the importance of all genes in the model. Eventually, 10 candidate genes were selected and used to construct another TreeGradientBoosting model. In this study, the C-indexes of the 10-gene-TreeGradientBoosting model in the training and validation cohorts were 0.776 and 0.848, respectively. The AUCs for the model predicting 1-, 3-, and 5-year survival were all over 0.75, indicating good performance and validating the substantial value of the GradientBoosting machine learning algorithm. The reason why the predictive performance in the validation cohort was better than in the training cohort is probably that the sample size in the validation cohort was small (n = 97), where the correctness of a single sample prediction can lead to large fluctuations in the overall results.

Risk scores were generated using the 10-gene-TreeGradientBoosting model to investigate the significance of the risk score in TME and tumor treatment. TME is composed of cellular and non-cellular components that allow the tumor to interact with its surroundings and play an important role in tumor progression and the immune system’s response to malignant tumors. Exploring TME using new technologies such as machine learning and image quantification has been a focus of scientific research^30^. In this study, the high-risk group had a higher percentage of immunophenotypes with low and medium cytotoxicity (36%, 42%) than the low-risk group (23%, 39%), and a lower percentage of high-cytotoxicity immunophenotype than the low-risk group (22vs. 38%, Fig. 4I). Tamborero et al.^19^ proposed that the strong antitumor immune response in samples with high-cytotoxicity immunophenotype might be due to immunogenic events such as ectopic expression of antigen, high mutation loads of DNA damage repair-associated genes^31^, alteration of antigen presentation mechanisms, and up-regulation of interferon. Tumors with medium-cytotoxicity immunophenotype are associated with high infiltration of suppressive immune cell populations, which suppress immune surveillance^32^ (e.g., M2 macrophage). Tumors with low-cytotoxicity immunophenotypes have disrupted the cancer-immunity cycle due to genetic alterations in the WNT– β-catenin pathway and upregulation of TGF-β and Hedgehog signaling pathways, which drive immune evasion^33,34^. In this study, TP53^35,36^ and PDE10A mutations (Fig. 2C), as well as enrichment of pathways such as WNT– β-catenin , TGF-β, and YAP/Hippo signaling pathways^37^ (Fig. 2D), led to the high frequency of low-cytotoxicity immunophenotypes and the tendency of tumor immune evasion in high-risk group. The low-risk group was associated with better antitumor immune responses due to immunogenic events such as microsatellite instability (Fig. 5A) and epigenetic inactivation of the MMR gene. As for the pathway enrichment analysis of differentially expressed genes between the two groups, some of the enriched pathways, such as the production of molecular mediators of immune response (Fig. 3A), complement activation^38^ (Fig. 3A), B cell receptor signaling pathway^39^ (Fig. 3A), cytokine-cytokine receptor interaction (Fig. 3B), IL-17 signaling pathway^40^ (Fig. 3B), were revealed to have complex relationships with TME and tumor immunity. Immune cell infiltration analysis using CIBERSORT.ABS (Fig. 4C), MCPCOUNTER (Fig. 4D), QUANTISEQ (Fig. 4E), XCELL (Fig. 4F), and ssGSEA (Fig. 4G) revealed that the low-risk group had a higher abundance of CD8 + T cells, T follicular helper cells, NK cells, and M1 macrophages and higher M1/M2 than the high-risk group, which were positively correlated with antitumor immune response^41^. Meanwhile, the low-risk group had lower MDSCs (Fig. 5G), TAM M2 scores (Fig. 5H) and Exclusion scores (Fig. 5E), and higher immune scores (Fig. 4A), indicating that the low-risk group had a higher level of antitumor immune cell infiltration. Comparison of the scores of 29 Fegs between the high-risk and low-risk groups (Fig. 4H), revealed that the low-risk group had significantly higher levels of antitumor immune markers, such as antitumor cytokines, co-activation molecule, checkpoint molecules, and T cells than the high-risk group. Figure 5D showed that the risk score was negatively correlated with 14 steps in Cancer-Immunity Cycle, including T cell recruitment, CD4+ T cell recruitment, CD8+ T cell recruitment, Th1 cell recruitment, and NK cell recruitment. Collectively, these findings indicated that the low-risk group had stronger antitumor immunity than the high-risk group.

Immune checkpoint genes (e.g., PDCD1 and CTLA4) were negatively correlated with the risk score (*P* < 0.05), and were highly expressed in the low-risk group. We speculated that selection pressure due to high immune cell infiltration in the low-risk group forced the tumors to present secondary changes like upregulation of inhibitory signaling PDCD1 pathway. Tamborero et al.^19^ proposed a similar opinion that selection pressure due to high-cytotoxicity immunophenotype led to upregulation of PD-L1/2, inhibitory chemokine, and JAK/STAT signaling pathways in tumors, which inhibited immune cell infiltration. Increased abundance of regulatory T cells and higher Dysfunction score in the low-risk group (Fig. 5I) could be partially attributed to secondary alterations arising from tumors resisting immune cell infiltration.

The cuproptosis-associated 10-gene-TreeGradientBoosting model predicted the prognoses of HNSC patients and generated risk scores associated with features like TME and tumor immunity. Nevertheless, the model also has some limitations. First, the construction and validation of the prognostic model were based on the TCGA and GEO databases, resulting in certain biases. Second, the study only employed retrospective data from the public database. Owing to the delay in data updates, it may not be possible for these public databases to accurately reflect the current state of HNSC in real-time. It is therefore imperative to conduct multi-center studies with large sample sizes in order to refine the model. Further prospective studies involving patients are required to validate the clinical utility of the model. Still, our study has substantial clinical implications.

## Supplementary Information

Supplementary Information 1.

Supplementary Information 2.

Supplementary Information 3.

Supplementary Information 4.

Supplementary Information 5.

Supplementary Information 6.

## Data Availability

The datasets generated and analyzed during the current study are available in the The Cancer Genome Atlas Program (TCGA, https: //portal.gdc.cancer.gov/) dataset and Gene Expression Omnibus (GEO, https://www.ncbi.nlm.nih.gov/geo/query/acc.cgi) dataset [GSE41613].

## Abbreviations

AUC: The area under the receiver operating characteristic curve
C-index: Harrell’s concordance index
CNV: Copy number variation
GB: Gradient Boosting
GDC: Genomic Data Commons
GEO: Gene Expression Omnibus
GO: Gene Ontology
HNSC: Head and neck squamous cell carcinoma
ICI: Immune checkpoint inhibition
KEGG: Kyoto Encyclopedia of Genes and Genomes
MCC: Maximum correlation criterion
ML: Machine learning
MSI: Microsatellite instability
OOB: Out-of-bag
OS: Overall survival
PPI: Protein–protein interaction
RF: Random Forest
SI: Stemness index
SVM: Support vector machine
TCGA: The Cancer Genome Atlas
TIDE: Tumor Immune Dysfunction and Exclusion
TME: Tumor microenvironment
TPM: Transcripts per thousand base million
