# Supplementary material for: Cuproptosis-related risk score based on machine learning algorithm predicts prognosis and characterizes tumor microenvironment in head and neck squamous carcinomas
Source: Sci Rep. 2023 Jul 22;13:11870. doi: 10.1038/s41598-023-38060-6 (PMC10363129; doi:10.1038/s41598-023-38060-6)
Supplement: Supplementary file 1 — Supplementary Information 1. [file 41598_2023_38060_MOESM1_ESM.docx]

Supplementary Table 1. Module, Class and Hyperparameters of Grid Search in Python for Each Model

| Algorithm | Module and class | Hyperparameters for grid search | Hyperparameters selected |
| --- | --- | --- | --- |
| FastSurvivalSVM | sksurv.svm.FastSurvi  valSVM | alpha:  2.0 ** [-12,-10,-8,……,8,10,12] | alpha=2.0, rank_ratio=1.0, max_iter=1000, tol=1e-5, random_state=0 |
| RandomSurvivalForest | sksurv.ensemble.Ran  domSurvivalForest | n_estimators:  [1,2,3,4,……,7,8,9,10,20,30,……,80,90,100,200,400,600,……,4600,4800,5000],  min_samples_leaf: [3,4,5,6,……,18,19,20] | n_estimators=6, max_depth=None, min_samples_split=6, min_samples_leaf=15, min_weight_fraction_leaf=0.0, max_features='sqrt', max_leaf_nodes=None, bootstrap=True, oob_score=False, n_jobs=4, random_state=None, verbose=0, warm_start=False, max_samples=None |
| TreeGradientBoosting | sksurv.ensemble.Gra  dientBoostingSurvival  Analysis | n_estimators:  [10,20,30,……,80,90,100,200,300,400,……,2800,2900,3000],  learning_rate:  [0.01,0.1,0.2,0.3,0.4,0.5] | loss='coxph', learning_rate=0.01, n_estimators=600, criterion='friedman_mse', min_samples_split=2, min_samples_leaf=1, min_weight_fraction_leaf=0.0, max_depth=3, min_impurity_split=None, min_impurity_decrease=0.0, random_state=None, max_features=None, max_leaf_nodes=None, presort='deprecated', subsample=1.0, dropout_rate=0.0, verbose=0, ccp_alpha=0.0 |
| ComponentwiseGradientBoosting | sksurv.ensemble.Co  mponentwiseGradient  BoostingSurvivalAnal  ysis | n_estimators:  [10,20,30,……,80,90,100,200,300,400,……,2800,2900,3000],  learning_rate:  [0.01,0.1,0.2,0.3,0.4,0.5] | loss='coxph', learning_rate=0.01, n_estimators=2000, subsample=1.0, dropout_rate=0, random_state=None, verbose=0 |
| CoxPHSurvival | sksurv.linear_model.  CoxPHSurvivalAnalys  is |  | alpha = 0，ties ='breslow'，n_iter = 100，tol = 1e-09，verbose = 0 |
