# Supplementary material for: Cuproptosis-related risk score based on machine learning algorithm predicts prognosis and characterizes tumor microenvironment in head and neck squamous carcinomas
Source: Sci Rep. 2023 Jul 22;13:11870. doi: 10.1038/s41598-023-38060-6 (PMC10363129; doi:10.1038/s41598-023-38060-6)
Supplement: Supplementary file 2 — Supplementary Information 2. [file 41598_2023_38060_MOESM2_ESM.docx]

Supplementary Table 1 The HR, 95% CI, and P-values of cuproptosis-related genes associated with survival

|  | HR | | 95% lower of HR | 95% upper of HR | P-values |
| --- | --- | --- | --- | --- | --- |
| CYCS | | 1.620432 | 1.261038 | 2.082252 | 0.000161 |
| PSMB5 | | 1.563772 | 1.212819 | 2.01628 | 0.000565 |
| COPZ1 | | 1.920798 | 1.309729 | 2.816968 | 0.000835 |
| RSL1D1 | | 1.599376 | 1.200794 | 2.130261 | 0.001322 |
| PFKP | | 1.310335 | 1.101588 | 1.558638 | 0.002267 |
| SLC25A39 | | 1.570502 | 1.164191 | 2.118619 | 0.003124 |
| MRPS23 | | 1.492717 | 1.137753 | 1.958426 | 0.003834 |
| MRPS7 | | 1.531777 | 1.140602 | 2.057107 | 0.004591 |
| COA6 | | 1.275603 | 1.07703 | 1.510788 | 0.00481 |
| NUBPL | | 1.4452 | 1.117888 | 1.868349 | 0.004947 |
| CDKN2A | | 0.921733 | 0.870768 | 0.975679 | 0.004979 |
| AASDHPPT | | 1.320716 | 1.083677 | 1.609602 | 0.005848 |
| SRP54 | | 1.428248 | 1.107762 | 1.841453 | 0.005972 |
| FDX1 | | 1.423125 | 1.102339 | 1.837261 | 0.006777 |
| NDUFB5 | | 1.31154 | 1.075373 | 1.599573 | 0.007421 |
| MRPS5 | | 1.46047 | 1.103 | 1.933792 | 0.008183 |
| STK11 | | 0.718644 | 0.560207 | 0.92189 | 0.009323 |
| MRPL21 | | 1.183637 | 1.03937 | 1.347929 | 0.011014 |
| TOP1MT | | 1.241381 | 1.049966 | 1.467692 | 0.011387 |
| COX17 | | 1.307776 | 1.062132 | 1.610232 | 0.011477 |
| CDC27 | | 1.404145 | 1.077878 | 1.829171 | 0.011876 |
| DOT1L | | 0.747083 | 0.594879 | 0.93823 | 0.012124 |
| SLC7A5 | | 1.155675 | 1.0302 | 1.296433 | 0.013612 |
| RPL19 | | 1.344168 | 1.062454 | 1.70058 | 0.013709 |
| ATIC | | 1.37298 | 1.06562 | 1.768993 | 0.014226 |
| PCBP2 | | 1.468566 | 1.079843 | 1.997222 | 0.014301 |
| TKT | | 1.221925 | 1.039483 | 1.436387 | 0.015127 |
| YARS2 | | 1.35211 | 1.059387 | 1.725716 | 0.015375 |
| EIF3I | | 1.528225 | 1.080652 | 2.161169 | 0.016455 |
| ZNF365 | | 1.212413 | 1.035785 | 1.419161 | 0.016501 |
| PHF5A | | 1.346703 | 1.053232 | 1.721946 | 0.017619 |
| ABCE1 | | 1.299505 | 1.041632 | 1.621218 | 0.020266 |
| RPS25 | | 1.298281 | 1.036025 | 1.626924 | 0.023368 |
| RRN3 | | 1.318213 | 1.037754 | 1.674469 | 0.023599 |
| SLC25A51 | | 1.303356 | 1.035143 | 1.641065 | 0.02421 |
| TRIM32 | | 1.212541 | 1.022696 | 1.437628 | 0.026535 |
| BUD31 | | 1.314347 | 1.028407 | 1.67979 | 0.028981 |
| WDR12 | | 1.284136 | 1.021148 | 1.614854 | 0.032439 |
| IMP4 | | 1.338932 | 1.024721 | 1.74949 | 0.032442 |
| MRPL17 | | 1.315185 | 1.021233 | 1.693749 | 0.033775 |
| MRPL30 | | 1.31086 | 1.020959 | 1.683079 | 0.033786 |
| RPL27 | | 1.31076 | 1.020577 | 1.683451 | 0.034048 |
| NDUFA8 | | 1.294315 | 1.019576 | 1.643085 | 0.034072 |
| GLS | | 1.183356 | 1.01227 | 1.383358 | 0.034598 |
| TIMM9 | | 1.267978 | 1.015887 | 1.582625 | 0.035787 |
| MDH2 | | 1.328512 | 1.017425 | 1.734716 | 0.036899 |
| RUVBL2 | | 1.310088 | 1.014188 | 1.69232 | 0.038657 |
| MRPS18A | | 1.322132 | 1.009095 | 1.732279 | 0.042802 |
| SHMT2 | | 1.231988 | 1.006693 | 1.507702 | 0.042898 |
| TSC22D2 | | 1.182201 | 1.005159 | 1.390425 | 0.043163 |
| ATP7B | | 1.233919 | 1.005364 | 1.514432 | 0.04431 |
| MRPS14 | | 1.343686 | 1.005078 | 1.796371 | 0.046135 |
| SSBP1 | | 1.333289 | 1.001787 | 1.774488 | 0.048586 |
| OXA1L | | 1.286148 | 1.000925 | 1.652649 | 0.049161 |
| NDUFV1 | | 1.230946 | 1.000091 | 1.515089 | 0.049899 |
