# Supplementary material for: Cuproptosis-related risk score based on machine learning algorithm predicts prognosis and characterizes tumor microenvironment in head and neck squamous carcinomas
Source: Sci Rep. 2023 Jul 22;13:11870. doi: 10.1038/s41598-023-38060-6 (PMC10363129; doi:10.1038/s41598-023-38060-6)
Supplement: Supplementary file 3 — Supplementary Information 3. [file 41598_2023_38060_MOESM3_ESM.docx]

Supplementary Table 2. Gene of top 20 MCC in STRING network

| Rank | Name | Score |
| --- | --- | --- |
| 1 | MRPS7 | 6736 |
| 2 | MRPS5 | 6000 |
| 3 | MRPL17 | 5893 |
| 4 | MRPS14 | 5892 |
| 5 | MRPL21 | 5890 |
| 6 | MRPL30 | 5886 |
| 7 | MRPS23 | 5760 |
| 8 | MRPS18A | 5042 |
| 9 | RPL27 | 895 |
| 10 | RPL19 | 890 |
| 11 | RPS25 | 856 |
| 12 | EIF3I | 754 |
| 13 | ABCE1 | 750 |
| 14 | SSBP1 | 733 |
| 15 | OXA1L | 146 |
| 16 | CYCS | 67 |
| 17 | NDUFB5 | 54 |
| 18 | NDUFA8 | 52 |
| 19 | NDUFV1 | 51 |
| 20 | WDR12 | 47 |
