# Supplementary material for: Cuproptosis-related risk score based on machine learning algorithm predicts prognosis and characterizes tumor microenvironment in head and neck squamous carcinomas
Source: Sci Rep. 2023 Jul 22;13:11870. doi: 10.1038/s41598-023-38060-6 (PMC10363129; doi:10.1038/s41598-023-38060-6)
Supplement: Supplementary file 4 — Supplementary Information 4. [file 41598_2023_38060_MOESM4_ESM.docx]

Supplementary Table 3 The AUC and 95% CI of different OS predictive models on 1-year, 3-years and 5-years survival

|  | 1 years (%) | | 3 years (%) | 5 years (%) |
| --- | --- | --- | --- | --- |
| FastSurvivalSVM | | 59.3(53.1-65.6) | 63.4(57.2-69.7) | 62.3(53.7-71.0) |
| RandomSurvivalForest | | 76.8(71.9-81.8) | 80.5(75.5-85.4) | 84.0(77.8-90.2) |
| TreeGradientBoosting | | 85.7(81.7-89.7) | 90.2(86.9-93.4) | 92.3(88.7-95.9) |
| ComponentwiseGradientBoosting | | 55.9(49.4-62.3) | 64.2(58.0-70.5) | 65.6(56.7-74.7) |
| CoxPHSurvival | | 68.4(62.7-72.0) | 72.2(67.5-77.7) | 74.8(67.3-79.5) |
