# Supplementary material for: Cuproptosis-related risk score based on machine learning algorithm predicts prognosis and characterizes tumor microenvironment in head and neck squamous carcinomas
Source: Sci Rep. 2023 Jul 22;13:11870. doi: 10.1038/s41598-023-38060-6 (PMC10363129; doi:10.1038/s41598-023-38060-6)
Supplement: Supplementary file 5 — Supplementary Information 5. [file 41598_2023_38060_MOESM5_ESM.docx]

Supplementary Table 4 The comparison on 1-year, 3-years and 5-years survival prediction between different OS predictive models

|  | 1 years | 3 years | 5 years |
| --- | --- | --- | --- |
| FastSurvivalSVM vs RandomSurvivalForest | < 0.001 | < 0.001 | < 0.001 |
|  |  |  |  |
| FastSurvivalSVM vs TreeGradientBoosting | < 0.001 | < 0.001 | < 0.001 |
|  |  |  |  |
| FastSurvivalSVM vs ComponentwiseGradientBoosting | 0.246 | 0.767 | 0.389 |
|  |  |  |  |
| FastSurvivalSVM vs CoxPHSurvival | 0.072 | 0.084 | 0.181 |
|  |  |  |  |
| RandomSurvivalForest vs TreeGradientBoosting | < 0.001 | < 0.001 | < 0.001 |
|  |  |  |  |
| RandomSurvivalForest vs ComponentwiseGradientBoosting | < 0.001 | < 0.001 | < 0.001 |
|  |  |  |  |
| RandomSurvivalForest vs CoxPHSurvival | 0.056 | 0.061 | 0.077 |
|  |  |  |  |
| TreeGradientBoosting vs ComponentwiseGradientBoosting | < 0.001 | < 0.001 | < 0.001 |
|  |  |  |  |
| TreeGradientBoosting vs CoxPHSurvival | < 0.001 | < 0.001 | < 0.001 |
|  |  |  |  |
| ComponentwiseGradientBoosting vs CoxPHSurvival | < 0.001 | 0.095 | 0.120 |
