# Supplementary material for: Cuproptosis-related risk score based on machine learning algorithm predicts prognosis and characterizes tumor microenvironment in head and neck squamous carcinomas
Source: Sci Rep. 2023 Jul 22;13:11870. doi: 10.1038/s41598-023-38060-6 (PMC10363129; doi:10.1038/s41598-023-38060-6)
Supplement: Supplementary file 6 — Supplementary Information 6. [file 41598_2023_38060_MOESM6_ESM.docx]

Supplementary Table 5 The feature importance of each gene

| Gene | Weight | Std |
| --- | --- | --- |
| MRPS14 | 0.037035 | 0.007186 |
| OXA1L | 0.03245 | 0.003549 |
| CYCS | 0.032347 | 0.011632 |
| NDUFB5 | 0.029157 | 0.005782 |
| EIF3I | 0.024823 | 0.003637 |
| RPL19 | 0.022493 | 0.003307 |
| MRPS23 | 0.019987 | 0.00661 |
| NDUFV1 | 0.019827 | 0.004754 |
| RPS25 | 0.019313 | 0.003221 |
| MRPS7 | 0.019251 | 0.00685 |
| SSBP1 | 0.011147 | 0.003846 |
| NDUFA8 | 0.009721 | 0.001052 |
| ABCE1 | 0.009649 | 0.001465 |
| MRPL21 | 0.007913 | 0.002517 |
| MRPS5 | 0.007859 | 0.002514 |
| MRPL17 | 0.006622 | 0.003353 |
| MRPL30 | 0.006011 | 0.000868 |
| RPL27 | 0.005017 | 0.000748 |
| MRPS18A | 0.004383 | 0.000929 |
| WDR12 | 0.000724 | 0.000355 |
